# Physiological and psychological responses to five-day fasting

**DOI:** 10.1371/journal.pone.0324929

**Published:** 2025-06-06

**Authors:** Albertas Skurvydas, Natalja Istomina, Ruta Dadeliene, Dovile Valanciene, Vaiva Hendrixson, Voldemaras Giedrimas, Asta Mockiene, Damian Luka Mialkowskyj, Daiva Majauskiene

**Affiliations:** 1 Department of Rehabilitation, Faculty of Medicine, Physical and Sports Medicine, Institute of Health Sciences, Vilnius University, Vilnius, Lithuania; 2 Education Academy, Vytautas Magnus University, Kaunas, Lithuania; 3 Faculty of Medicine, Institute of Health Sciences, Vilnius University, Vilnius, Lithuania; 4 Faculty of Law, Vilnius University, Vilnius, Lithuania; 5 Faculty of Medicine, Institute of Biomedical Sciences, Vilnius University, Vilnius, Lithuania; 6 Institute of Physiology and Pharmacology, Medical Academy, Lithuanian University of Health Sciences, Kaunas, Lithuania; 7 Department of Physiology, Faculty of Medicine, Biochemistry, Microbiology and Laboratory Medicine, Institute of Biomedical Sciences, Vilnius University, Vilnius, Lithuania; Sarich Neuroscience Research Institute, AUSTRALIA

## Abstract

The objective of this study was to examine the variations in adipokines, myokines, inflammation indicators, glucose, insulin, and ketones in the body over a 5-day fasting period. Additionally, the study aimed to investigate the underlying factors contributing to changes in body mass index (BMI) and fat mass. These factors included blood markers, participants’ healthy lifestyle habits, emotional intelligence, personality traits, impulsivity, overall well-being, and subjective happiness. The study involved 42 women with an average age of 49.8 years (± 9.3 years). The following indicators were measured: leptin, adiponectin, TNF-alpha, BDNF, irisin, IL-6, insulin, glucose levels, and ketone bodies. Various assessments were utilized, including the Physical Activity Questionnaire, the Brunel Mood Scale (BRUMS-LTU), the Schutte Self-Report Emotional Intelligence Test, the 10-item Perceived Stress Scale, and the Big Five personality traits. The results showed that fasting led to substantial reductions in body mass, waist circumference, leptin levels, glucose, and insulin levels, while simultaneously increasing ketone bodies. Basal energy expenditure decreased, but participants experienced improvements in mood, with increased vigor and reduced tension. Although markers of inflammation rose, the concentration of irisin declined, while levels of BDNF and adiponectin remained unchanged. Moreover, a greater reduction in fat mass was associated with higher pre-fasting well-being, emotional intelligence, and lower levels of tension and impulsivity. Conversely, the loss of lean mass was linked to neuroticism and higher levels of impulsivity, particularly concerning pre-fasting tension levels.These findings suggest that psychological factors may impact fasting outcomes, emphasizing the need for personalized fasting strategies.

## Introduction

Intermittent fasting has garnered significant attention in recent years due to its profound impact on various biological processes. Currently, there is compelling evidence that intermittent fasting increases the expression of antioxidant defences, deoxyribonucleic acid (DNA) repair, protein quality control, mitochondrial biogenesis, autophagy, and down-regulates inflammation [1–3]. It effectively serves as a preventive measure against neurodegenerative diseases, obesity, insulin resistance, dyslipidemia, hypertension, and inflammation [4–8].

Intermittent fasting exhibits a neurotrophic effect, specifically an increase in brain-derived neurotrophic factor (BDNF) levels in the brain cortex, thereby promoting synaptic plasticity and neurogenesis [3,4,9,10]. Research indicates that BDNF plays a crucial role in mediating the positive effects of energetic challenges such as intense exercise and fasting on cognition, mood, cardiovascular function, and peripheral metabolism [4]. The metabolic ‘switch’ from glucose, derived from liver glycogen stores, to fatty acids and their ketone metabolites in adipose cells, occurring during fasting, seems to serve as a crucial catalyst for both brain-intrinsic and peripheral organ-derived signals that enhance synaptic plasticity, neurogenesis, memory, and learning. Brain-intrinsic extracellular signals involve the excitatory neurotransmitter glutamate and the neurotrophic factor BDNF, while peripheral signals may encompass the liver-derived ketone 3-hydroxybutyrate and the muscle cell-derived protein irisin [11]. A recently published study shows that an increase in BDNF concentration can improve brain health and overall well-being during aging [12]. Our study showed that 10 weeks of regular yoga practice improved balance under both single- and dual-task conditions and enhanced motor task learning, with these changes being associated with an increased BDNF level. However, yoga had no effect on cognitive function in older adults [13].

Fasting causes the change of various hormones, including insulin, glucagon, glucagon-like peptide-1, catecholamines, ghrelin, and fibroblast growth factor 21. The levels of adipokines, such as leptin and adiponectin, are also adjusted, along with cell stress-induced cytokines, such as tumour necrosis factor-alpha, and exerkines, such as interleukin-6 and irisin [14]. Adiponectin is crucial for maintaining systemic nutrient availability and energy balance during energetic stresses, such as fasting [14]. In their review, Townsend and Steinberg [14] highlight the role of adiponectin in activating AMP-activated protein kinase (AMPK), a central regulator of energy homeostasis. Activation of AMPK by adiponectin leads to increased glucose uptake and fatty acid oxidation in tissues such as skeletal muscle and liver, thereby improving insulin sensitivity. This pathway underscores adiponectin’s significant role in maintaining metabolic balance and its potential therapeutic implications for metabolic disorders. It has been determined that elevated circulating irisin is associated with a decreased cardiometabolic risk and improved cardiorespiratory fitness [15,16]. Moreover, irisin may influence the molecular mechanisms of vascular aging, including inflammation, oxidative stress, and epigenetics [14]. Irisin also plays a role in the browning of white adipose tissue, and its levels may increase during physical activity and fasting [17].

Despite the aforementioned studies, there is a dearth of research on the fluctuations in body mass composition (lean and fat mass), blood concentrations of glucose, ketones, insulin, adipokines (leptin and adiponectin), as well as inflammation indicators (TNF-alpha and IL-6), and myokines (BDNF and irisin) during intermittent fasting. Therefore, the objective of our study was to examine the variations in adipokines, myokines, inflammation indicators, glucose, insulin, and ketones in the body over a 5-day fasting period, and to investigate the underlying factors contributing to changes in body mass index (BMI) and fat mass, including blood markers, participants’ healthy lifestyle habits, emotional intelligence, personality traits, impulsivity, well-being, and subjective happiness..Our hypothesis suggests that a five-day fasting period would enhance fat oxidation, reduce levels of insulin and leptin, increase adiponectin levels, and improve metabolic flexibility. Additionally, we expected it to modulate inflammatory markers such as IL-6 and TNF-alpha, thereby reducing inflammation. We also anticipated positive effects on mood and energy perception, along with increases in BDNF and irisin, due to their roles in metabolic regulation, neuroprotection, and cardiovascular health. Therefore, we believe that five-day fasting could serve as a safe and effective caloric restriction strategy that promotes metabolic flexibility, brain and cardiovascular health, and psychological well-being.

## Methods

### Participants

The study was conducted from September 19–23, 2022, involving 42 healthy women with an average age of 49.8 years (± 9.3). Based on the specified statistical parameters (p < 0.01, Cohen’s d > 0.8, and statistical power = 0.8), the required sample size to achieve sufficient statistical power for detecting significant changes is approximately 38 participants. Participants in this study were selected according to specific inclusion and exclusion criteria to ensure sample homogeneity and result reliability. The inclusion criteria required that participants be women aged 40–60 years, generally healthy. We did not specifically assess the participants’ menopausal status. However, since the participants were not engaged in shift work, their sleep-wake cycles aligned with a typical daytime schedule, resulting in minimal circadian disruption. They needed to have maintained a stable body weight for at least three months prior to the study and not to be taking any medications that could directly affect metabolism, such as corticosteroids or thyroid hormones. Additionally, participants had to be able and willing to adhere to the fasting protocol and study procedures; they must not have used any weight loss interventions in the three months prior. The exclusion criteria eliminated individuals with severe chronic diseases, including uncontrolled diabetes, cardiovascular diseases, or cancer, as well as those taking medications known to influence metabolic or inflammatory markers. Women undergoing hormone replacement therapy or experiencing recent hormonal changes were also excluded. The study was conducted according to the guidelines of the Declaration of Helsinki and was approved by Lithuanian Bioethics Committee (No.2021/11-1393-866). All participants provided written informed consent before inclusion.

### Measurements

**Anthropometric measurements** were conducted to assess body composition, including fat mass, muscle mass, lean body mass, body mass index (BMI), water mass, waist-to-hip ratio (WHR), basal energy expenditure (BEE), and distribution of visceral fat (DWF). These measurements were taken using the BC-300 body composition analyzer (Tanita, Jawon, South Korea). The participating women also had their height, systolic and diastolic blood pressure, and waist circumference measured

**Sociodemographic indicators** included age, gender, education, and job type reported by the participants.

Physical activity (PA) was assessed using the long version of the **International Physical Activity Questionnaire** (IPAQ) [18] adapted into Lithuanian [19]. This questionnaire covers four activity domains: occupational PA (paid employment and voluntary work), transport-related PA, domestic PA, and recreational PA. The IPAQ items assess the frequency of PA (reported in the number of days; “During the last 7 days, on how many days did you do...?”) and average duration of PA per day (reported in hours and minutes; “How much time did you spend on one of those days doing...?”) in these specific PA domains. Total weekly PA was estimated by weighting the time spent on each activity according to its intensity by its metabolic equivalent (MET) energy expenditure. The METs of vigorous, moderate, and low-intensity activities were 8.0, 4.0, and 3.3, respectively.

**Emotional intelligence** (EI) was assessed through the Schutte Self-Report Emotional Intelligence Test (SSREIT) [20]. SSREIT has been translated and used in previous research in Lithuania [21–23] SSREIT is a 33-item questionnaire divided into four subscales as follows: Perception of emotions (10 items), Ability to deal with one’s own emotions (9 items), Ability to deal with emotions of others (8 items), and Use of emotions (5 items). The items are designed to be answered on a five-point scale ranging from 1 = strongly disagree to 5 = strongly agree. Total scores ranged from 33 to 165, with higher scores indicating higher ability in the area of EI.

**The Brunel Mood Scale**-LTU (BRUMS-LTU) [24,25] adapted from Terry et al., [26] was used to assess responses about mood. The 24-item BRUMS- LTU scale has six subscales with four items each (i.e., Tension Items: nervous, anxious, worried, panicky; Depression Items: unhappy, miserable, depressed, downhearted; Anger Items: bitter, angry, annoyed, energetic; Vigour Items: energetic, active, lively, alert; Fatigue Items: exhausted, tired, worn out, sleepy; and Confusion Items: mixed up, muddled, uncertain, confused). The participants responded using a 5-point Likert scale (0 = not at all, 1 = a little, 2 = moderately, 3 = quite a bit, and 4 = extremely), with the total possible subscale scores ranging from 0 to 16 points. The time frame was right now, i.e., “How do you feel right now?”. The 24 items condensed into six subscale scores were treated as scale variables. The BRUMS-LTU has demonstrated satisfactory internal consistency, with Cronbach’s α coefficients ranging from.74 to.90 for the six subscales.

**Personality traits** (BIG-5) [27] were assessed using the Big Five Inventory (BFI) adapted into Lithuanian [28]. The 44-item scale allows for an efficient and flexible assessment of five personality dimensions when there is no need for more differentiated measurement of individual facets. Participants rate each BFI item on a 5-point scale ranging from 1 (strongly disagree) to 5 (strongly agree) and the scale scores are computed as the participant’s mean item response (i.e., adding all items scored on a scale and dividing by the number of items on the scale). The scale scores are calculated as the sum of respective items.

**Perceived stress** was assessed through the 10-item Perceived Stress Scale (PSS-10) [29] adapted into Lithuanian [30]. PSS-10 is a 10-item self-reported questionnaire designed to assess the extent to which the individual has perceived situations in their life as unpredictable, uncontrollable and overloading over the past month. It consists of 10 questions designed to be answered on a five-point scale ranging from 0 = never to 4 = very often. Total scores ranged from 0 to 40, with higher scores indicating a higher perceived stress level.

**Smoking and alcohol consumption** were assessed by asking the respondents to indicate their smoking habits on a scale of 1–4, where 1 is “I have never smoked”; 2 is “I smoke occasionally”; 3 is “I smoke every day”; 4 is “I used to smoke, but I quit”. Alcohol consumption was assessed on a scale of 1–7, where 1 is “I don’t drink at all”; 2 is “Several times a year”; 3 is “Once a month”; 4 is “Several times a month”; 5 is “Once a week”; 6 is “Several times a week”; and 7 is “Daily”.

**Eating breakfast and overeating** were assessed by asking the respondents to indicate their eating habits. Eating breakfast was evaluated on a scale of one to three, where 1 is “no”; 2 is “sometimes”; and 3 is “yes”). Overeating was also assessed using a scale of one to three, where 1 is “no”; 2 is “rarely”; and 3 is “often”).

The **Life Satisfaction Index** (living standards) was determined by asking the question “Are you satisfied with your life?” with response options on a 10-point scale ranging from 1 = very dissatisfied to 10 = very satisfied.

The **Happiness index** was assessed by asking the question “Are you happy in life?” with response options on a 10-point scale from 1 = very unhappy to 10 = very happy.

### Testing of organokines in blood samples

The blood samples were collected in the morning (7:00–9:00 AM) in a fasted state on both the first and last days of the fasting program to account for circadian variations in metabolic markers, ensuring that the measurements are comparable. Venous blood was drawn into tubes that contained a blood coagulation activator and separator gel. These samples can be stored at temperatures between 4–8°C for up to 24 hours without freezing, before serum separation. Therefore, the samples were transported in a vertical position at 4°C to Vilnius University Hospital Santaros Clinics on the same day.Upon arrival at the laboratory, the blood samples were centrifuged at 1000g for 10 minutes at 4°C. The serum was then separated into coded tubes and frozen at −20°C until biochemical analysis could be performed. To maintain the integrity of the samples, multiple freeze-thaw cycles were avoided, and all samples were analyzed within a specified timeframe to prevent potential degradation. The concentrations of leptin, adiponectin, TNF-alpha, BDNF, irisin, IL-6, and insulin in the serum were determined using the antibody sandwich enzyme-linked immunosorbent assay (ELISA) method, utilizing Elabscience ELISA detection kits from China. The analysis was conducted following the manufacturer’s protocol. The Thermo Scientific Multiscan SkyHigh from Singapore was used for plate incubation, shaking, and optical density (OD) reading at 450 nm, with correction at 630 nm. The sensitivities of the ELISA assays used were as follows: leptin 0.05 ng/mL, adiponectin 0.5 µg/mL, TNF-alpha 0.1 pg/mL, BDNF 0.005 ng/mL, irisin 0.1 ng/mL, IL-6 0.1 pg/mL, and insulin 0.1 µIU/mL. To ensure assay precision, the intra-assay coefficient of variation (CV%) was kept below 5%, while the inter-assay CV% remained below 7%. All measurements were performed in duplicate, and standard curves with known concentrations were generated for each analyte to ensure accuracy.Capillary blood glucose levels were measured using the GLUCOCARD S glucometer, which has a detection range of 1.1 to 33.3 mmol/L and a precision of ±5%. The concentration of ketone bodies (β-hydroxybutyrate) in capillary blood was measured using the FREESTYLE OPTIUM NEO ketone meter, with a detection range of 0–8 mmol/L and a precision of ±0.1 mmol/L. To ensure the reliability of biochemical analyses, internal quality controls at low, medium, and high concentration levels were included in each assay. Standard curves were generated for ELISA measurements, and duplicate readings were conducted. If the coefficient of variation (CV%) exceeded the acceptable threshold, the sample was reanalyzed.

### Procedures

**The preparation stage** lasted four weeks. During this time, the participants were recommended to eat three times a day without any snacking between meals (breakfast, lunch, and dinner). The recommended time for breakfast was 8:00 AM, and for dinner 6:00 PM. No specific meal plan was provided and compliance with specified recommendations was not monitored.

**The fasting stage lasted for 5 days**. The participants arrived at the retreat centre at the agreed time and did not consume any food for 120 hours. They could drink only water and had to adhere to the daily schedule: waking up at 7:00 AM and going to bed at 10:00 PM. We aimed to create lifestyle conditions during the five-day fasting period that closely resembled everyday healthy living, incorporating optimal physical activity, sauna sessions, and massage to enhance overall well-being. Every morning the participants engaged in a 50-minute facilitated morning exercise session. Each day they walked 5–7 km outdoors with walking intensity at 50–70% of the maximum heart rate. Every day they attended educational and psychological sessions led by specialists. On the second day of fasting, the participants received a 60-minute full-body relaxation massage, and on the third day, they took a humid 70°C sauna with three rounds of 15–20 minutes each. During the fasting stage, the participants’ condition was monitored by a physiscian.

The glucose level was measured three times a day for each participant during the fasting stage of the program. On the first, third, and fifth days of fasting, ketone bodies (beta-hydroxybutyrate) were measured too.

At the **end of the fasting period**, the participants gradually returned to a normal eating routine according to the diet plan prepared by a nutritionist. During this period, they adhered to a special diet plan, which included food consumption recommendations and appropriate food quantities.

At the beginning and the end of the fasting period, the participants completed a questionnaire, and anthropometric measurements, and blood samples were taken.

### Data analysis

First, the normality of the data distribution was assessed using the Shapiro-Wilk test, confirming that all variables followed a normal distribution. To evaluate the differences in means before and after fasting among women, a paired samples t-test was performed, and Cohen’s d was calculated to determine the effect size. For categorical variables, a chi-square test was applied to compare distributions before and after fasting, with the corresponding p-values reported. Correlation analyses utilized Pearson’s correlation coefficient (r), which was interpreted according to standard guidelines. The internal consistency of the questionnaires was assessed using Cronbach’s α, with values above 0.7 considered acceptable. All statistical analyses were conducted using IBM SPSS Statistics (version 22; IBM Corp., Armonk, NY, USA). Results are presented as means with standard deviations (M ± SD) for continuous variables and as percentages for categorical data. Exact p-values are reported for statistical significance where applicable.

## Results

80% of the participating women had a university education. 83% of the participants did sitting and standing tasks at work. On average, women rated their life happiness at 7.7 (±1.8) and the cost of living at 8.3 (±1.3) out of 10 points. The BMI of the women was 30.8 ± 5.6 kg/m^2^.

Half of the women participating in the study exercise and maintain a high level of physical activity (3189.9 MET ± 800.2 min/week). 56% of the participants do not smoke, 22% do not drink alcohol, 61% have breakfast and 22% do not overeat. Notably, 72% of the participants (30 women) had lost weight over the past five years, though they did not experience any weight loss in the three months leading up to the start of the experiment. Among the women who had lost weight within this five-year period, the average weight loss was 10.5 ± 7.8 kg, and this process took approximately 4.7 ± 3.1 months on average. The main baseline descriptive indicators are presented in Table 1.

**Table 1:** Average baseline descriptive values (including standard deviation).

	Evaluation	SD
Number	42	
Women, %	100	
Age, years	49.8	9.3
Higher education, %	83	
Happiness, points	7.7	1.8
Living standards, points	8.3	1.3
KMI, kg/m^2^	30.8	5.6
Exercise, %	50	
Exercise, MET min/week	3189.9	800.2
No smoking, %	56	
No alcohol drinking, %	22	
Breakfast eating, %	61	
No overeating, %	22	
Weight loss over the past 5 years, % of women	72	
Weight lost over a period of 5 years, kg	10.5	7.8
Time taken to lose weight, months	4.7	3.1
Sleeping time, h	7.69	0.65
Bedtime, h	23.16	1.01
Emotional intelligence, pt	129.6	9.9
Impulsivity total, pt	62.1	4.8
Extroversion, pt	36.9	4.7
Conscientiousness, pt	38.4	5.8
Agreeableness, pt	40.5	4.5
Neuroticism, pt	28.1	6.0
Openness, pt	49.3	8.7

**Note.** SD – standard deviation.

The research findings reveal that during a 5-day fasting period, the body mass decreased by 4.25 ± 0.76 kg (4.8 ± 0.8%) (p < 0.0001), the fat mass decreased by 1.07 ± 1.5 kg (3.7 ± 3.1%) (p < 0.01), the lean body mass (LBM) decreased by 3.18 ± 1.7 kg (5.4 ± 1.8%) (p < 0.0001) (Table 2).

**Table 2:** The effects of a 5-day fast on blood indicators, body composition, and blood pressure before and after the fasting period.

	Before		After		After/before %		p-value	Cohen d
	Average	SD	Average	SD	Average	SD		
Leptin, ng/mL	4.22	2.1	1.55	1.3	45.2	20.1	0.0001	1.6
Adiponectin, µg/mL	35.4	8.5	37.6	8.4	110.1	20.9	0.108	−0.26
Leptin/adiponectin	0.14	0.07	0.04	0.02	36.2	12.8	0.0001	1.9
BDNF, ng/mL	22.7	7.5	21.8	6,4	98.5	7.2	0.18	0.13
Irisin, ng/mL	8.4	2.5	6.7	1.7	90.1	17.4	0.015	0.79
TNF-alpha, pg/mL	31.5	14.1	38.5	11.5	125.9	21.1	0.01	−0.54
IL-6, pg/mL	1.57	0.45	2.31	0.77	152.2	35.4	0.0001	−1.2
Insulin, µIU/mL)	4.97	1.1	1.61	0.47	33.5	18.1	0.0001	3.9
Glucose, mmol/l	5.2	1.2	3.7	0.54	69.2	18.1	0.0001	1.6
Ketone body, mmol/l	0.82	0.51	5.2	0.91	625.5	71.4	0.0001	−5.9
Waist circumference, cm	96.1	8.5	90.1	7.1	87.1	12.2	0.0001	0.81
Body mass, kg	88.9	10.1	84.6	9.7	95.2	0.82	0.0001	0.51
Fat mass, kg	32.6	8.7	31.5	7.1	96.3	5.7	0.018	0.14
Water mass, kg	40.6	7.1	38.2	5.4	94.5	1.8	0.0001	0.38
Lean body mass, kg	56.3	8.1	53.1	7.1	94.6	1.5	0.0001	0.45
Fat mass, %	36.2	5.4	36.6	5.4	101.4	4.1	0.17	−0.01
BEE, kcal/day	1322.8	145.1	1287.1	104.1	97.5	0.8	0.0001	0.37
BEE, kcal, kg/day	15.1	1.1	15.5	1.8	102.5	1.4	0.0003	−0.33
SBP, mm/Hg	125.7	8.4	126.5	8.9	101.8	3.4	0.085	−0.01
DBP, mm/Hg	79.4	7.1	80.5	5.1	98.9	3.5	0.35	−0.27
WHR	0.91	0.07	0.92	0.08	100.6	1.1	0.11	−0.11
DWF, cm²	138.3	44.5	147.8	54.3	104.7	14.2	0.094	−2.1

**Note.** BEE – basal energy expenditure, SBP – systolic blood pressure, DBP – diastolic blood pressure, WHR – waist-to-hip ratio, DWF – distribution of visceral fat, p – the level of marginal significance within a statistical hypothesis test.

**Fig 1:**
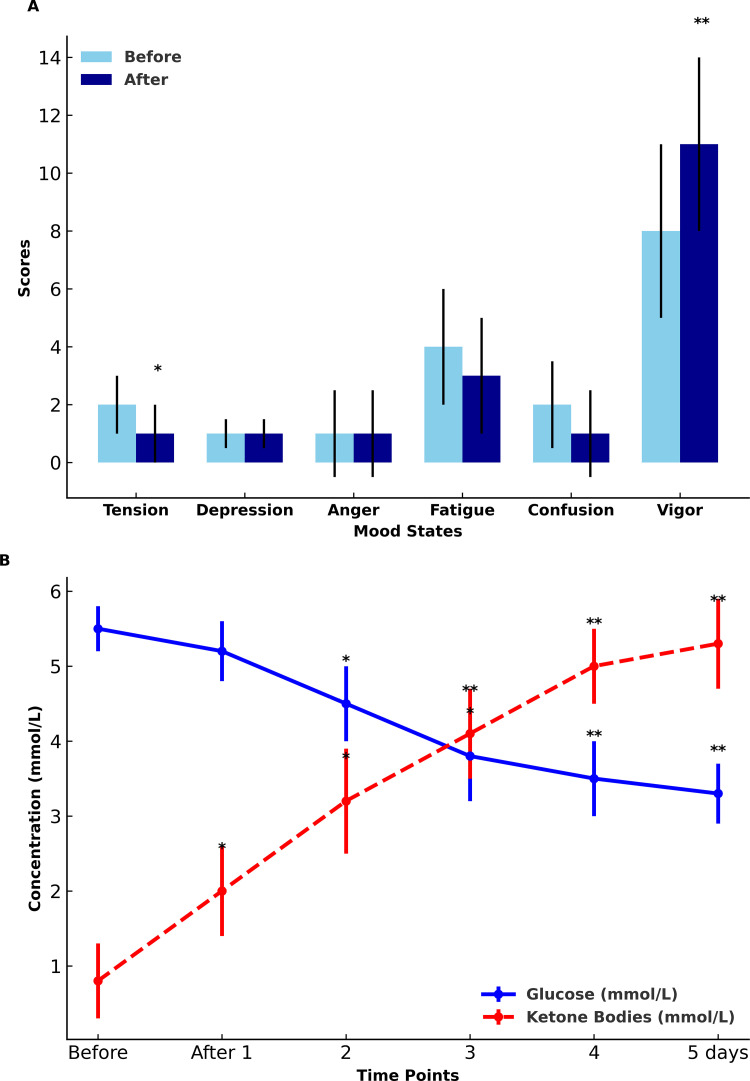
Changes in glucose and ketone bodies (A) and mood indicators (B) before and after five days of fasting.

Notably, the basal energy expenditure (BEE) decreased by 2.5 ± 1.2% (p < 0.01). However, the relative BEE (kcal/kg/day) increased significantly. There was a pronounced decrease in leptin, insulin, and glucose levels (p < 0.0001) (Table 2), while ketone bodies increased approximately fivefold (p < 0.0001) (Fig 1A).

Furthermore, irisin concentration showed a slight but significant decrease (p < 0.01), TNF-alpha increased (p < 0.01) with no significant change in BDNF or adiponectin concentration (p > 0.05). Systolic and diastolic blood pressure (SBP and DBP) did not exhibit significant changes over the 5 fasting days (p > 0.05). Waist circumference decreased by 6.6 ± 5.5 cm (6.34 ± 4.2%) (p < 0.0001). It is noteworthy that the waist-to-hip circumference ratio and the area of internal/visceral fat did not change significantly while the fat area even tended to increase. Among all mood indicators, vigour increased (p < 0.001), and tension decreased (p < 0.01) after five days of fasting (Fig 1B).

It should be noted that the decrease in fat mass and LBM over the five days of fasting correlated significantly with the initial adiponectin concentration, corresponding to r = −0.75, p < 0.0001 and r = 0.79, p < 0.0001 (Table 3). Thus, the higher the adiponectin level before fasting, the more the fat mass decreased and the less the LBM decreased due to the fasting. Additionally, the higher the pre-fasting concentration of TNF-alpha, the more the BMI decreased during fasting (r = −0.58; p < 0.0001). There is an interesting observation that the lower the initial tension and impulsivity level, and the higher the Emotional Intelligence (EI), the more the fat mass decreased over the five days of fasting (in all cases, the correlation coefficient was statistically significant, p < 0.0001) (Table 3).

**Table 3:** Correlation Coefficients with Statistical Significance (n = 42).

	Adiponectin	BDNF	Irisin	Leptin	Insulin	TNFalpha	Tension	EI	Impulsivity	Neuroticism
**Fat, after/before, %**	−0.75***	−0.37*	−0.12	−0.05	−0.12	−0.15	0.58***	−0.59***	0.58***	0.11
**LBM, after/before, %**	0.79***	0.41**	0.09	0.11	0.45**	−0.22	−0.64***	0.22	−0.12	−0.59***
**LBM, after/before, %**	0.79***	0.41**	0.09	0.11	0.45**	−0.22	−0.64***	0.22	−0.12	−0.59***

Note.*- p < 0.05; ** p < 0.01; ***- p < 0.0001.

Conversely, the higher neuroticism and tension levels, the more LBM decreased during fasting, with respective correlation coefficients of r = −0.59, p < 0.0001, and r = −0.63, p < 0.0001. We observed a parallel decrease in LBM and BEE (r = 0.94, p < 0.0001) during the fasting period. Conversely, there was a lower decrease in BEE with a higher decrease in fat mass (r = −0.93, p < 0.0001).

## Discussion

Our study’s key findings reveal that significant changes occurred during the 5-day fasting period, particularly in glucose and ketone metabolism, insulin and leptin levels, and waist circumference. These changes were statistically significant (p < 0.0001, Cohen’s d ≥ 0.8) and indicate metabolic adaptation alongside enhanced fat oxidation. We also observed an increase in IL-6 levels, which suggests a potential inflammatory response to fasting. In contrast, TNF-alpha levels increased (p = 0.01, Cohen’s d = −0.54), reflecting the complexity of inflammation during this period. Interestingly, we noted a pronounced increase in vigor (p < 0.001, Cohen’s d = 0.94), indicating an improvement in psychological well-being and energy perception associated with fasting. These results are consistent with the expected physiological responses, as prolonged fasting typically induces shifts in energy utilization, hormonal regulation, and inflammatory processes. However, contrary to our expectations, the levels of adiponectin and BDNF remained unchanged, despite their known roles in metabolic and neurotrophic adaptations. Additionally, we found that irisin, which is recognized for its cardiovascular protective effects, decreased (p = 0.015, Cohen’s d = 0.79), suggesting a potential reduction in its beneficial role during the fasting period.

Throughout the five-day fasting period, there was a notable reduction in body mass and waist circumference, as expected, a significant decrease in leptin and glucose concentration, and an increase in ketone body concentration in the blood. This aligns with the findings of other researchers [31–34]. There is evidence that fasting elevates serum ketone levels, providing an alternative fuel source for the brain and other organs, and ketone bodies can act as signalling metabolites, influencing gene expression, inflammation, and oxidative stress [33]. However, the BEE decreased, and the reduction in fat mass was less pronounced compared to LBM. This aligns with the findings of Ezpeleta et al. [32]. In our study, SBP and DBP did not decrease during fasting contrary to what other researchers have shown [31,32,34]. Moreover, as a result of fasting, mood indicators improved: vigour increased, and tension decreased. This aligns with the findings of other researchers [7,35]. However, as Solianik et al. [36] showed, fasting for 48 hours induced greater psychological and physiological stress in females.

Surprisingly, the anticipated increase in BDNF concentrations and adiponectin did not manifest, although numerous studies suggest that fasting elevates BDNF concentration [3,4,11]. However, as illustrated by Gibbons et al. [37], fasting for 20 hours resulted in a 9-fold increase in ketone body delivery to the brain but had no impact on BDNF in peripheral circulation at rest. BDNF facilitates favourable effects of energetic challenges, such as intense exercise and fasting, on cognition, mood, cardiovascular function, and peripheral metabolism [4]. Other studies also indicate that fasting increases adiponectin levels [14,31,38], although in our case adiponectin remained unchanged and this finding was rather unexpected. However, during 5 days of fasting, inflammation indicators increased and irisin concentration in the blood decreased. Furthermore, circulating irisin levels correlate with a reduction in cardiometabolic risk and an enhancement in cardiorespiratory fitness [15,17]. Thus, intermittent fasting appears to have a minimal or no effect on key inflammatory markers, but further research is needed to validate these initial findings [39]. One possible explanation for the unexpected finding that BDNF and adiponectin concentrations did not increase, despite the reduction in body mass and waist circumference, lower glucose, leptin, and insulin levels, and an overall enhancement in mood over the five-day fasting period, could be related to the duration and intensity of the fasting period. Five days of fasting might not have been sufficient to trigger a significant increase in BDNF and adiponectin levels, as these organokines can respond more slowly to changes in metabolic conditions compared to other markers. Additionally, the decrease in fat mass was less notable compared to lean body mass, which might indicate that the adipose tissue did not undergo substantial changes to elevate adiponectin production significantly. Furthermore, individual variations in metabolic responses and hormonal regulation could play a role. Factors such as baseline levels of BDNF and adiponectin, genetic predispositions, and differences in how participants’ bodies adapt to fasting could influence the outcomes. Also, the overall enhancement in mood suggests positive psychological effects of fasting, which might not directly correlate with immediate changes in BDNF and adiponectin levels. These neurotrophic and adipokine responses might require a longer period of sustained metabolic and physiological alterations to manifest significantly. Our previous research has shown that after just a few weeks of yoga training, the concentration of BDNF in the blood can increase by approximately twofold [13]. Additionally, it has been well established that higher levels of BDNF contribute to improved brain function and enhance the brain’s resilience against early aging [12]. Research indicates that the expression of BDNF increases in response to fasting [2–4]. This upregulation contributes to improved neuroplasticity, cognitive function, and stress resilience [2–4]. The underlying mechanism involves the production of ketone bodies and a reduction in oxidative stress, which together activate pathways that enhance neuronal survival and synaptic plasticity [2]. We can conclude that the duration of fasting, the extent of fat mass reduction, individual metabolic variations, and the time needed for these organokines to respond could explain why BDNF and adiponectin concentrations did not increase as expected.

Intriguingly, the reduction in fat mass was the most strongly linked to the initial high level concentration of adiponectin in the blood, as well as to a higher EI and lower levels of tension and impulsivity values compared to those recorded before the fasting. However, higher levels of neuroticism and tension correlated with a greater decrease in LBM during fasting. The adiponectin/leptin ratio showed a significant (p < 0.05) inverse correlation with BMI, waist circumference, measures of adiposity, and insulin levels among all participants at baseline [40]. Furthermore, it has been clearly demonstrated that adiponectin plays a crucial role in maintaining systemic energy balance [14]. Our research only suggests the hypothesis that emotional state may be associated with fat reduction mechanisms. However, further long-term studies are necessary to validate this hypothesis.

## Limitations

The main limitation of our study is the absence of a control group. To address this, we focused only on objective and reliable indicators, such as blood markers. Additionally, all study participants were women with an average age of around 50 years. This presents another limitation, as the findings may not be applicable to younger women or men. Furthermore, it is challenging to fully control fasting conditions. We ensured that the participants’ daily activities closely mirrored their usual routines, with fasting being the only significant intervention. To maintain a balanced lifestyle, participants engaged in moderate-intensity physical activity, attended a sauna session, and received a massage once during the study. Naturally, these factors may have influenced our results. In interpreting our findings, it is essential to consider the characteristics of our study population. The average body mass index (BMI) of the participants was 30.8 kg/m^2^, and a significant portion of the group—80%—held a university or college degree. Another limitation of our study is that 30 out of the 42 women had attempted to lose weight within the past five years, although none had done so in the three months leading up to the study. As a result, the physiological response to weight loss may have been diminished in those with prior attempts. Future studies should consider this factor to examine how the body’s response to fasting is affected by the number of previous fasting attempts, comparing first-time and repeated attempts. Additionally, prolonged fasting (five days in our study) requires greater psychological effort and may pose potential health risks. In contrast, intermittent or short-term fasting appears to be a safer and more sustainable strategy for promoting health benefits [34]. Finally, we acknowledge that a key limitation of our study is that correlation coefficients do not imply causation. For instance, while emotional intelligence was linked to fat mass reduction over 5 days of fasting, this relationship should be interpreted with caution, as it does not establish a direct causal link. Additionally, it remains challenging to determine how an individual’s psychological state might be causally related to the specific physiological mechanisms underlying weight loss and whether psychological factors serve as a cause, a consequence, or both.

## Conclusion

Although our study has limitations, the findings provide objective and meaningful data. They demonstrate that a five-day fasting period induces significant metabolic and psychological adaptations, though some biomarkers showed unexpected variations. During the fasting period, we observed decreases in body mass, waist circumference, glucose, leptin, and insulin levels, accompanied by improvements in mood. However, the anticipated increases in BDNF (brain-derived neurotrophic factor) and adiponectin did not occur. Instead, we found that pro-inflammatory markers increased, while irisin levels decreased, suggesting a complex physiological response that goes beyond simple metabolic adaptation. A key insight from our study is the relationship between changes in body composition and psychological traits. We found that a greater reduction in fat mass was associated with higher emotional intelligence and lower levels of tension and impulsivity. Conversely, loss of lean body mass correlated with higher neuroticism and increased tension prior to fasting. These findings imply that psychological predispositions may influence the outcomes of fasting, highlighting the importance of personalized fasting approaches.

## Supporting information

S1 File Raw data used in analyses. (XLSX)
